# Thank you to the *Journal of the Medical Library Association* reviewers in 2019

**DOI:** 10.5195/jmla.2020.929

**Published:** 2020-04-01

**Authors:** Katherine G. Akers

**Affiliations:** Editor-in-Chief, *Journal of the Medical Library Association*, JMLA@journals.pitt.edu, http://orcid.org/0000-0002-4578-6575

## Abstract

The *Journal of the Medical Library Association (JMLA)* sincerely thanks the 220 peer reviewers in 2019 who helped vet and improve the quality of work published in our journal.

The *Journal of the Medical Library Association (JMLA)* sincerely thanks the 220 peer reviewers in 2019 who helped vet and improve the quality of work published in our journal.

*JMLA* is always looking to expand our pool of reviewers who can critically comment on any topic of research or practice in health sciences librarianship. If you are interested in serving as a peer reviewer for *JMLA*, please send your CV to the editor-in-chief at jmla@journals.pitt.edu.

## 2019 *JOURNAL OF THE MEDICAL LIBRARY ASSOCIATION* REVIEWERS

Elizabeth Aitken

Kristine M. Alpi, AHIP

Katherine Anderson

Katelyn Angell

Marilia Y. Antunez, AHIP

Thea Atwood

Caitlin Bakker, AHIP

Michael Bales

Jill Barr-Walker

Michelle B. Bass, AHIP

Danielle Becker

Christopher William Belter

Skye Bickett, AHIP

Brooke L. Billman, AHIP

Lindsay E. Blake, AHIP

Catherine Boden

Sarah Bonato

Jill Boruff, AHIP

Alexandria Leigh Brackett, AHIP

Wichor Bramer

Marci Brandenburg

Ellen Brassil, AHIP

Emily Brennan

Tara Brigham

Adrienne Brodie

Stewart Brower, AHIP

Heather Brown

Roy Eugene Brown, AHIP

Krystal Bullers, AHIP

John Bullion

Christopher Burns

Nicole Capdarest-Arest, AHIP

Alexander J. Carroll, AHIP

Tallie Casucci

Susan Cavanaugh

Thane Chambers

Robin Champieux

Liza Chan

Marina Chilov

Natalie Clairoux

Heather Coates

Keith W. Cogdill, AHIP

Jamie Conklin

Nicole Contaxis

Marisa Conte, AHIP

Jean-Paul Courneya

Jill Crawley-Low

Andrew Creamer

John W. Cyrus

Sandy De Groote, AHIP

Ariel Deardorff

Diana Delgado, AHIP

Michelle Demetres

Robin Desmeules

Jennifer Dinalo

Daniel Dollar

Kathel Dunn

Martha Earl, AHIP

Erin RB Eldermire

Jonathan Eldredge, AHIP

Marina Englesakis

Keith Engwall, AHIP

Electra Enslow

Lisa Federer, AHIP

Rita Fleming-Castaldy

David Flynn

Margaret Jane Foster, AHIP

Jacqueline Freeman

Melissa C. Funaro

Emily J. Glenn

Abigail Goben

Alexandra Gomes, AHIP

Kelsey Grabeel, AHIP

Adelia Grabowsky

Vera Granikov

Jean Gudenas, AHIP

Gale G. Hannigan, AHIP

Ardis Hanson

Susan Maria Harnett, AHIP

Teresa Hartman

R. Brian Haynes

Margaret Henderson, AHIP

Rachel Hinrichs, AHIP

Elizabeth G. Hinton, AHIP

Carmen Howard

Carol L. Howe

Matt Hoy, AHIP

Shanda Hunt

Emily J. Hurst, AHIP

Holly Jackson

Rebecca Jacob

Sarah T. Jewell

Phill Jo, AHIP

Emily M. Johnson, AHIP

Timothy Johnson

Ellen Justice, AHIP

Kellie N. Kaneshiro, AHIP

Jill R. Kavanaugh, AHIP

Susan Keller

Andrea Ketchum

Sujin Kim

Michele Klein-Fedyshin, AHIP

Lorie Andrea Kloda, AHIP

Molly Knapp, AHIP

Rachel Amelia Santose Koenig

Laura Koppen

Laura Kuo, AHIP

Erica Lake, AHIP

Mariana Lapidus

Fred Willie Zametkin LaPolla

Deborah L. Lauseng

Janna C. Lawrence, AHIP

Gregory Laynor

Anne M. Linton, AHIP

Elizabeth R. Lorbeer, AHIP

Diane Lorenzetti

Ya-Ling Lu

Irene (Rena) Machowa Lubker, AHIP

Jinxuan Ma

Mark MacEachern

Tara R. Malone

Derek Marshall

Heather J. Martin, AHIP

Lisa Mastin

Karen McElfresh, AHIP

Bethany S. McGowan

Lisa McGuire

Dina J. McKelvy, AHIP

Sandra McKeown

Misa Mi, AHIP

Elizabeth Moreton

Whitney Mortensen

Bethany Myers, AHIP

Sawyer Newman

Annie Nickum, AHIP

Tyler Nix

Hannah Friggle Norton, AHIP

Melanie J. Norton

Kelly O’Brien

Marilyn Oermann

Eugenia Opuda

Rob O’Reilly

Lisa Palmer, AHIP

Janet Papadakos

Robin Parker

Melanie Parlette-Stewart

Emily Patridge, AHIP

Donald Pearson, AHIP

Robert Perret

Gerald J. Perry, AHIP, FMLA

Keith Pickett

T. Scott Plutchak, AHIP, FMLA

Ariel FitzGerald Pomputius

Kimberly R. Powell

Susan Powelson

Zahra Premji

Katie Prentice, AHIP

Livia Puljak

Alexandria Quesenberry

Bart Ragon

Gurpreet Kaur Rana

Marcia Rapchak

Rebecca Raszewski, AHIP

Melissa Ratajeski, AHIP

Rebecca Raworth

Robyn Reed, AHIP

Melissa L. Rethlefsen, AHIP

Megan Rosenbloom, AHIP

Amanda Ross-White, AHIP

Stephanie Roth, AHIP

Alexandra Sarkozy

Cathy Sarli, AHIP

Kate Saylor

Carolyn Schubert

Stephanie Schulte

April Joy Schweikhard, AHIP

Amanda Scull

Barbara Sen

Robert Shapiro II

Claire Sharifi

Tracy C. Shields, AHIP

Mary Shultz

John Siegel, AHIP

Erin M. Smith

Judith Smith

Edwin Vincent Sperr Jr., AHIP

Stuart Spore

Susan Steelman, AHIP

Elizabeth Stellrecht

Sean Stone

Judy Carol Stribling, AHIP

Alisa Surkis

Stephanie M. Swanberg, AHIP

Natalie Tagge

Michele R. Tennant, AHIP

Nicole Theis-Mahon, AHIP

Annie Thompson

Holly Thompson

Lorraine Toews

Lauren Topper

Efren Macanlalay Torres Jr.

Ivana Truccolo

Rose L. Turner

Andrea Twiss-Brooks

Ana Ugaz

Marcy Vana

Emily Vardell

Kimberly Vardeman

Megan von Isenburg, AHIP

Stacey Elizabeth Wahl

Amanda Wanner, AHIP

Terrie R. Wheeler

Judith Ann Wiener

Jeff D. Williams, AHIP

Gloria Willson

Joe Wu

Lin Wu, AHIP

Jingfeng Xia

Lauren M. Young, AHIP

## 

**Katherine G. Akers, PhD**, JMLA@journals.pitt.edu, http://orcid.org/0000-0002-4578-6575, Editor-in-Chief, *Journal of the Medical Library Association*

